# Cellular Lactate Spectroscopy Using 1.5 Tesla Clinical Apparatus

**DOI:** 10.3390/ijms231911355

**Published:** 2022-09-26

**Authors:** Adrian Truszkiewicz, Dorota Bartusik-Aebisher, Jolanta Zalejska-Fiolka, Aleksandra Kawczyk-Krupka, David Aebisher

**Affiliations:** 1 Department of Photomedicine and Physical Chemistry, Medical College of The University of Rzeszow, University of Rzeeszów, 35-310 Rzeszów, Poland; 2 Department of Biochemistry and General Chemistry, Medical College of The University of Rzeszow, University of Rzeszów, 35-310 Rzeszów, Poland; 3 Department of Biochemistry, Faculty of Medical Sciences in Zabrze, Medical University of Silesia, 40-055 Katowice, Poland; 4 Center for Laser Diagnostics and Therapy, Department of Internal Medicine, Angiology and Physical Medicine, Medical University of Silesia in Katowice, 41-902 Bytom, Poland

**Keywords:** lactate, magnetic resonance spectroscopy, 1.5 Tesla field

## Abstract

Cellular lactate is a key cellular metabolite and marker of anaerobic glycolysis. Cellular lactate uptake, release, production from glucose and glycogen, and interconversion with pyruvate are important determinants of cellular energy. It is known that lactate is present in the spectrum of neoplasms and low malignancy (without necrotic lesions). Also, the appearance of lactate signals is associated with anaerobic glucose, mitochondrial dysfunction, and other inflammatory responses. The aim of this study was the detection of lactate in cell cultures with the use of proton magnetic resonance (^1^H MRS) and a 1.5 Tesla clinical apparatus (MR OPTIMA 360), characterized as a medium-field system. In this study, selected metabolites, together with cellular lactate, were identified with the use of an appropriate protocol and management algorithm. This paper describes the results obtained for cancer cell cultures. This medium-field system has proven the possibility of detecting small molecules, such as lactate, with clinical instruments. ^1^H MRS performed using clinical MR apparatus is a useful tool for clinical analysis.

## 1. Introduction

The measurement of lactate belongs to metabolomics, which investigates cell physiology in healthy and diseased in vitro or in vivo frameworks. Recently, progress in high-resolution analytical detection has dramatically increased the possibility of metabolite measurements and allowed researchers to become familiar with the previously unexplored field of metabolomics. Although these high-resolution methods, such as chromatographic, mass spectrometric, and nuclear magnetic resonance, allowed for metabolomics capturing using body fluids or tissue samples, not all metabolomics studies require this depth of coverage. In a clinical scenario, the challenge is to use non-invasive measurements without complicated sample preparation. In addition to this, with clinical routines, the complexity of the samples resulted in an increase in the time for sample preparation and analysis, higher costs, and an increase in errors arising from time and sample preparation changes.

Proton magnetic resonance spectroscopy (^1^H MRS) enables non-invasive information about the biochemical composition of selected tissues to be collected from the human body. Usually, ^1^H MRS is an in vivo measurement based on the detection of ^1^H signals from substances contained in the tumor and, thus, is often used in cancer diagnostics. The result of the measurement is a profile of metabolites in the tissue. ^1^H MRS allows researchers to determine the quality and quantity of chemical compounds that have arisen from metabolic processes in healthy or cancer tissues in a non-invasive manner and without the need to administer contrast agents into the tissue. As a result of the ^1^H MRS measurements, a spectrum is obtained, in which peaks represent cellular metabolites. Briefly, hydrogen atoms in the tissue are surrounded by other atoms, which affect their chemical shift. The areas under the individual peaks (for a specific substance) correspond to the concentrations of these substances in the tested tissue.

The quality of the different metabolites that are visible in the ^1^H MRS spectrum depends on the hardware and software of the MR system. The field strength of the MR system is of particular importance because the spectral resolution of the spectrum increases with a higher field strength [1,2]. Additionally, different values for echo times (TE) are used to obtain peaks from specific metabolites [1,2].

**Figure 1:**
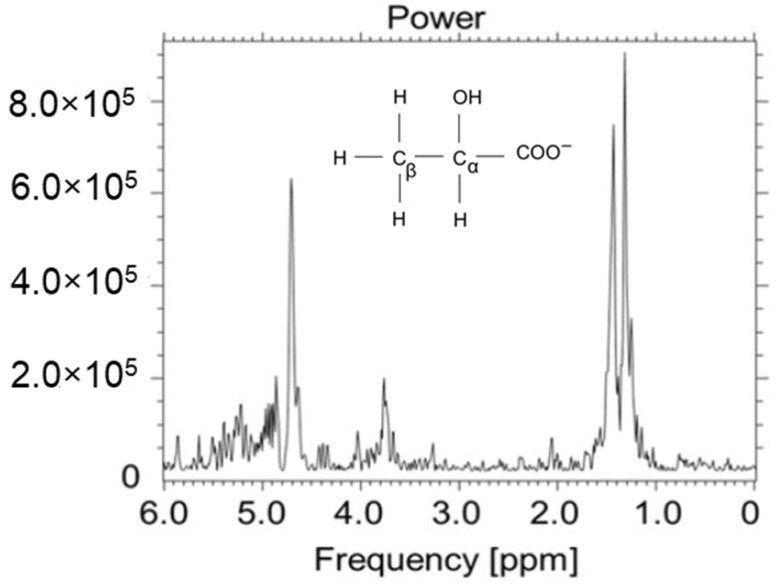
Lactate (Figure 1) has two resonances in the ^1^H spectrum: the first (β) at δ = 1.30 ppm, and the second (α) at δ = 4.08 ppm. The doublet is the result of the interaction of lactate proton peaks with the J-coupling (7 Hz). The second resonance (α) is a 1:3:3:1 quartet. The signal for TE = 144 ms is below the baseline, while for TE = 288 ms, it is above this line.

Lactate (Figure 1) is one of the many cellular metabolites presented in bodily tissue [1]. Knowledge on lactate concentration is fundamental for a thorough understanding of changes in cellular metabolism. Cell metabolism describes the chemical reactions which result from the conversion of nutrients into energy, as well as proteins, nucleic acids, or lipids. The adaptive abilities of neoplastic cells, as well as the complexity of the processes taking place in them, cause that neoplastic disease to resist treatment using conventional methods. The recognition of the relationship between the survival of cancer cells and changes in their metabolism has resulted in new possibilities for influencing cancer cells through specific metabolic changes [2]. It should be noted that the metabolism of a cancer cell may be heterogeneous, and the impact on it may also have consequences for healthy cells. Some of the current metabolism-based cancer therapies show that cancer therapy can be safely administered. The presence of lactate indicates energy transformations in the tested samples and is an important marker of anaerobic glycolysis. Medium-field MR apparatus has the ability to help with conducting research in the field of cell cultures. Selected metabolites can be identified with the use of an appropriate protocol and management algorithm. In the present study, we investigated the MRS detection of a small lactate concentration in cell culture using 1.5 Tesla MR clinical apparatus. Low-field lactate spectroscopy guarantees conditions that are the same or similar to clinical conditions when spectra are collected in vivo [3]. Cellular metabolomic data are highly challenging to collect for research. Metabolomics offers the promise that, in the future, the biochemical analysis of the entire path from genotype to phenotype will be measured and explored for new insights into biology and medicine [3,4,5,6,7].

Mass spectrometry (LC-MS or GC-MS)-based lactate detection is the most sensitive technique for the simultaneous analysis of a large number of compounds. This type of detection requires labeled standards for every single experiment. NMR complements cellular lactate detection by MS. An important additional feature of NMR is that it is quantitative and capable of providing absolute levels for the detected compounds when the appropriate techniques are used. MRS refers to the use of the MR phenomenon to determine the relative concentrations of specific molecules in the sample under investigation. The key output of MRS is an MR spectrum which graphically displays the detected signals as a function of their temporal frequencies. MRS provides unique information about the cellular substrates and their function in health and disease. MRS is a noninvasive method for measuring tissue content. The main challenge of using MRS for molecule detection (other than with water) is the low biological concentrations. For MRI, the hydrogen atoms within the bulk water within the tissue serve as an immense resource of protons. The biological abundance of hydrogen from water within tissue is approximately 65 M. Although bulk water provides an ample signal for imaging and allows for high spatial resolution (e.g., 1 mL voxel size), the detection of bulk water is not the motivation for MRS. ^1^H MRS experiments rely on the hydrogen atoms in the biologically important small molecules that are typically in millimolar concentrations, many orders of magnitude less than the concentration of hydrogen atoms in bulk water.

In this study, we present ^1^H MRS as a powerful approach for investigating lactate measurements in cancer cell culture. When conventional metabolomics methods are tuned to maximize the quantity of chemical diversity in the samples (after preparation), we address our methodology to detect lactate. To address this problem, we developed a coil that is specifically optimized to enable the quantification of lactate. We show that this approach enables lactate to be detected using 1.5 Tesla MRI apparatus.

## 2. Results

The procedure included the scanning of the samples in the mentioned OPTIMA 360MR system (GEMS, Germany).

### 2.1. Examination of Lactate as a Standard towards Its Search in the MRS Spectrum of Cell Culture

**Figure 2:**
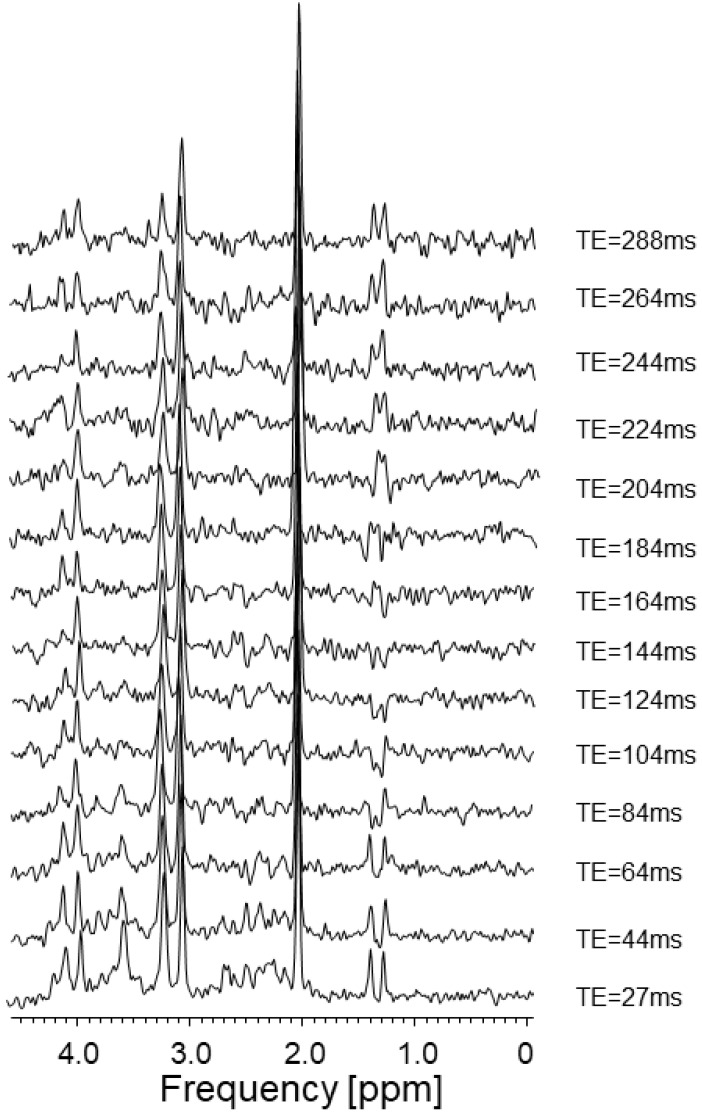
Evolution of the lactate signal as a function of TE time. The characteristic lactate peaks were around 1.3–1.4 ppm (own elaboration).

Phantom head spectra were collected with a head coil, as presented in Figure 2. As there are considerable difficulties when purchasing the fluid for filling the brain spectroscopy phantom for testing purposes, we decided to use C_3_H_6_O_3_ lactic acid for the coil. A higher number of measurement points made it possible to accurately visualize the evolution of the lactate peaks in the TE time domain. Firstly, the MRS results showed a dependence on the TE values. Selecting the TE was a critical factor in determining the appearance of the MRS. T_2_ relaxation decreases the transverse magnetization throughout the time of the three RF pulses, up to the TE, and also throughout the duration of the data acquisition. The TE values simplify both the baseline and the pattern of the peaks [8].

**Figure 3:**
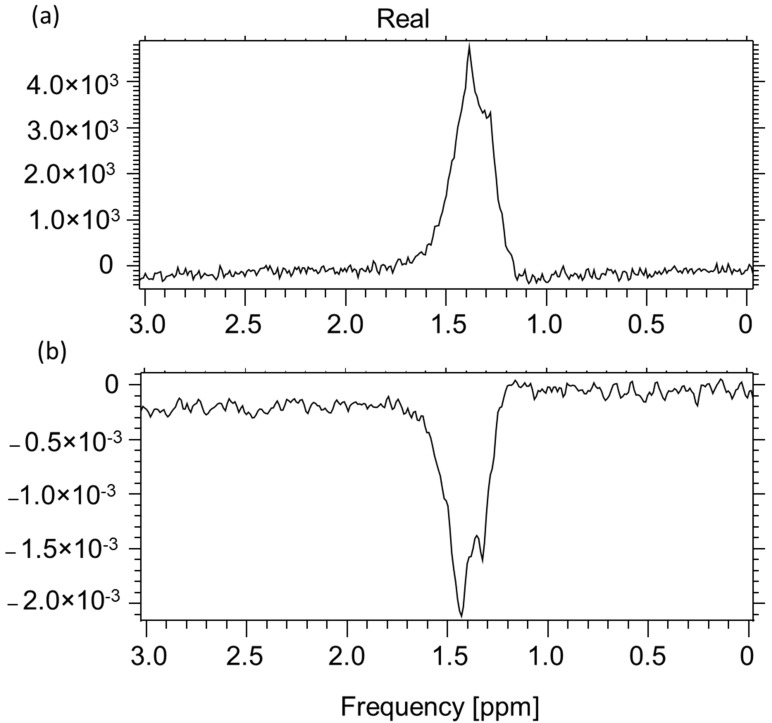
Lactic acid. (**a**) The spectrum presented in the upper part of the image was made for the echo time TE = 288 ms, NEX = 128, total number of scans: 128, sequence name: PROBE-P, (**b**) while for the lower spectrum: TE = 144 ms, NEX = 8, total number of scans: 128, sequence name: PROBE-P. The test performed with the experimental coil is shown in Figure 2. Voxel size: 8 × 8 × 8 mm (own elaboration).

The MRS result for the lactate aqueous solution as a function of TE is shown in Figure 3. The differences in the “Real” components of the spectrum for the time TE = 144 ms and TE-288 ms are clearly visible.

### 2.2. MRS Spectroscopy of RPMI-1640 Culture Fluid

The composition of the RPMI-1640 medium is presented in Table 1. The MRS test at a magnetic field of 1.5 T is a low-resolution test due to the properties of the phenomenon itself. In addition, low concentrations of RPMI-1640 ingredients mean that this study can only be analyzed to a limited extent and only retrospectively. Nevertheless, in the context of this article, it can be said with certainty that the culture medium is lactate-free. It is noteworthy that the high glucose content was visualized—it is particularly visible in the spectral range of 3.2–3.9 ppm, 4.6 ppm, 4.8 ppm, and 5.2 ppm.

**Figure 4:**
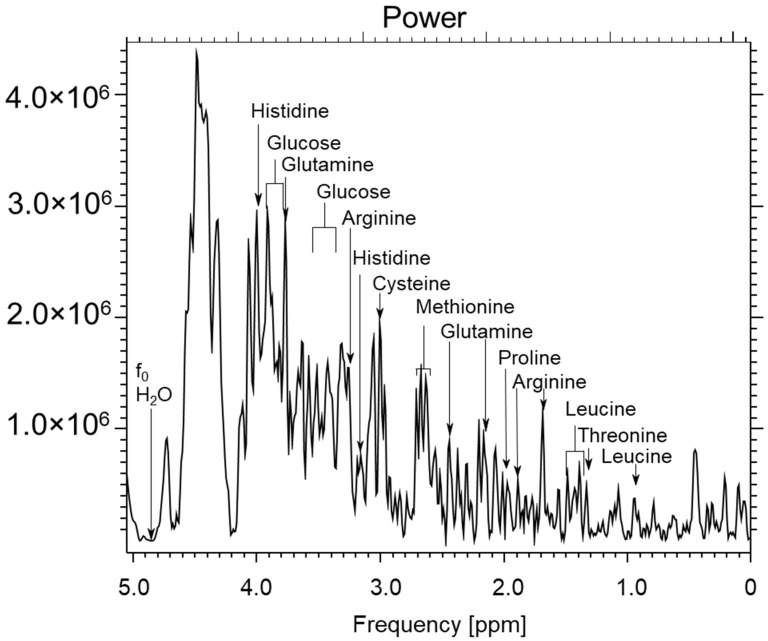
The results for scanning the culture medium, RPMI1640, using the 1.5 T system GEMS type Optima 360 MR, TE = 28, TR = 1500 ms, NEX = 8, total number of scans: 128, sequence name: PROBE-P. Voxel size: 8 × 8 × 8 mm (own elaboration).

**Figure 5:**
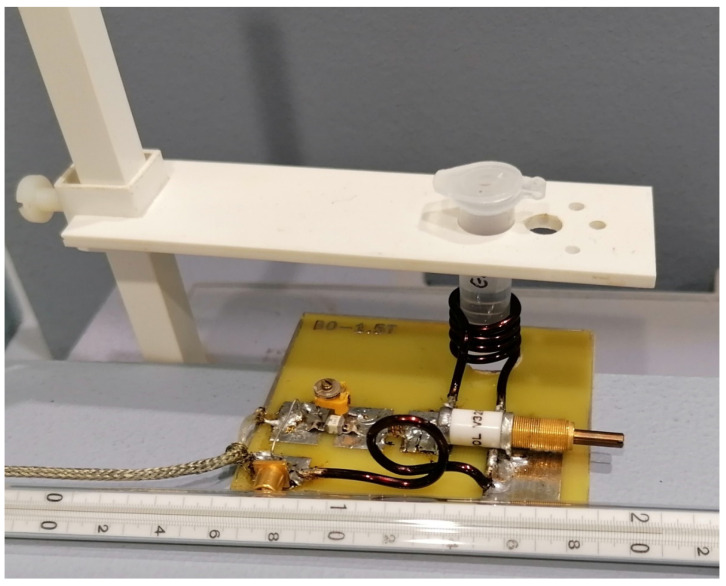
Glucose test (2 mL H_2_O + 50 mg Glucose).

Figure 4 shows the studies of RPMI cell culture medium used for the cultivation of the MCF7 and A549 cell lines. The remaining signals are derived from the compounds contained in the fluid. The spectrum comes from an experimental coil which is not described in this article. Figure 5 shows the setup for the glucose test (2 mL H_2_O + 50 mg Glucose).

At around 4.63 and 4.85, there should be a double and a single signal. Unfortunately, this is the range of water suppression, and thus all signal descriptions involve a lot of uncertainty. Parts of the useful signals are suppressed precisely because of water suppression.

### 2.3. A549 and CRL2314 Cell Culture Study

**Figure 6:**
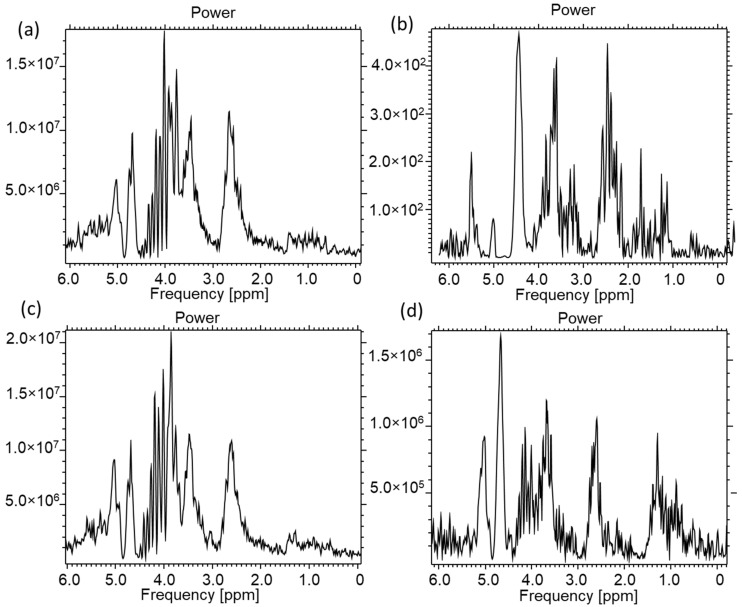
A549 cell culture spectroscopy, performed using the Optima 360MR clinical system, prod. GEMS. (**a**) TE = 28, TR = 4000 ms, NEX = 8, total number of scans: 128, sequence name: PROBE-P, (**b**) TE = 144, TR = 4000 ms, NEX = 8, total number of scans: 128, sequence name: PROBE-P, (**c**) TE = 35 ms, TR = 6000 ms, NEX = 8, total number of scans: 128, sequence name: PROBE-P, (**d**) TE = 288 ms, TR = 6000 ms, NEX = 8, total number of scans: 128, voxel size for all spectra: 8 × 8 × 8 mm (own elaboration).

Figure 6 contains four images resulting from the spectroscopic scanning of the A549 cell culture. The test cells were not subjected to additional processing due to the need for their further growth. The aim of the study was to record the signal from the tested object in its natural environment. The test was carried out at different values for TE and TR time by repeatedly changing the shimming settings manually. With such a small facility, these clinical systems, which are perfectly adapted to patient applications, do not always allow for optimal settings. The acquisition of the spectroscopic spectra was carried out at a temperature of 22 °C, which forced the related correction. A value of 4.85 ppm was used as the water point. In the vicinity of this point, the manifested water peak is clearly visible—it was suppressed by the methods contained in the MRS sequence itself, but due to its properties, it was also necessary to use the methods contained within the SAGE software.

**Figure 7:**
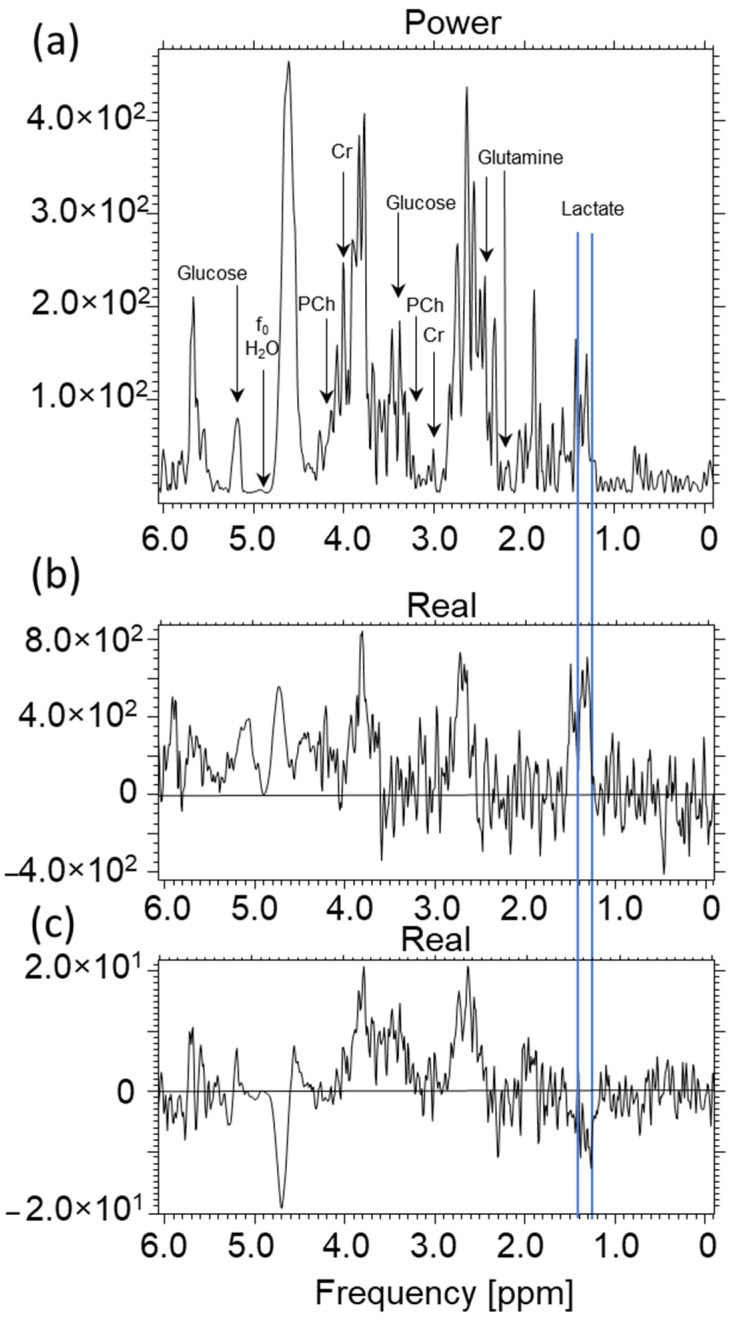
A549 cell culture spectroscopy, performed on the Optima clinical system prod. GEHC, (**a**) spectrum in Power mode TE = 144 ms, TR = 4000 ms, NEX = 8, total number of scans: 128, sequence name: PROBE-P, (**b**) component of the Real spectrum TE = 288 ms, TR = 4000 ms, NEX = 8, total number of scans: 128, sequence name: PROBE-P, (**c**) component of the Real spectrum TE = 144 ms, TR = 4000 ms, NEX = 8, total number of scans: 128, sequence name: PROBE-P. Voxel size for all spectra: 8 × 8 × 8 mm (own elaboration).

**Figure 8:**
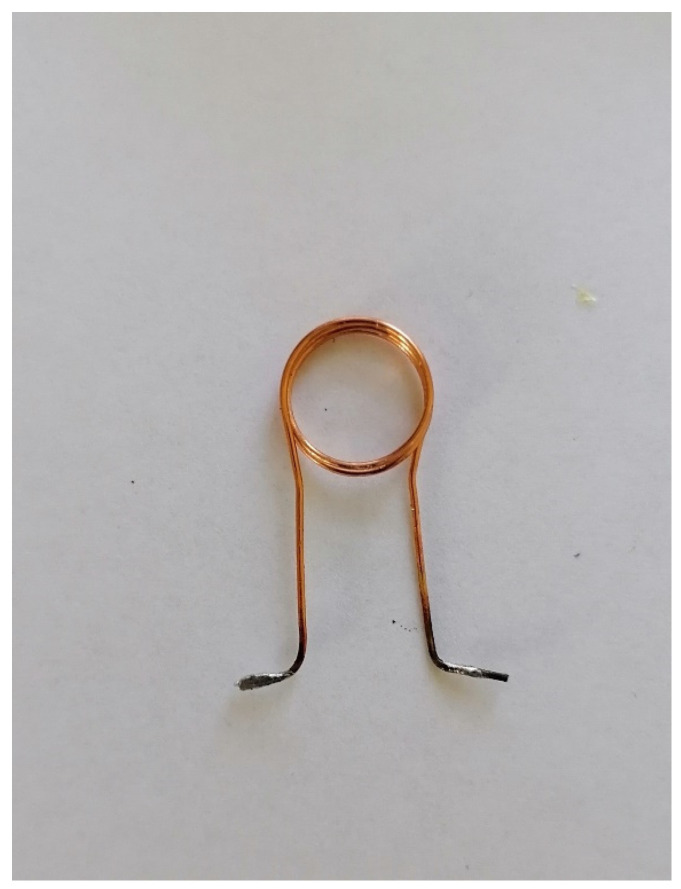
The appearance of the L1 inductor of the receiving coil.

The characteristic lactate peak manifests itself in the spectroscopic images. Lactate is the ionic form of lactic acid, and the product of anaerobic glycolysis is the result of anaerobic respiration. This characteristic doublet, for which the chemical shift is around 1.3 ppm, can be visualized by tests that use two different echo times: TE = 144 ms and TE = 288 ms. This makes it possible to distinguish lactates from lipids and macromolecules, the signals of which occur in this region of the spectroscopic scale. The amplitude spectrum does not show the dependencies resulting from the sign (resulting from the phase of the signal). In order to recognize this feature, it is necessary to analyze the real component of the signal. In Figure 7b, for the lower part, the real component diagram for TE = 144 ms is presented, while Figure 7c shows the real spectrum component for TE = 288 ms. It is clearly visible that the lactate signal has a negative phase for TE = 144 ms, which confirms that, in the amplitude plot, the peaks marked as lactates are specific to these metabolites. A test performed at TE = 288 ms shows this doublet in the “Real” window above the axis. The spectrum comes from an experimental coil not described in this article. Both of the coils used for testing are single-channel receiving coils. They do not require coupling with the head coil as they themselves are connected to the MR system via the coil seat. The receiving circuits differ from each other by the L1 coil. Its value is selected on a similar level, but it is made of thin wire and therefore has a shorter length. It was re-tuned after soldering (Figure 8).

The aim of the study was the detection of lactate in cell cultures. The construction of the various experimental coils seemed to be of secondary importance. The description of one of them was presented for the order and rules governing the writing of scientific texts.

**Figure 9:**
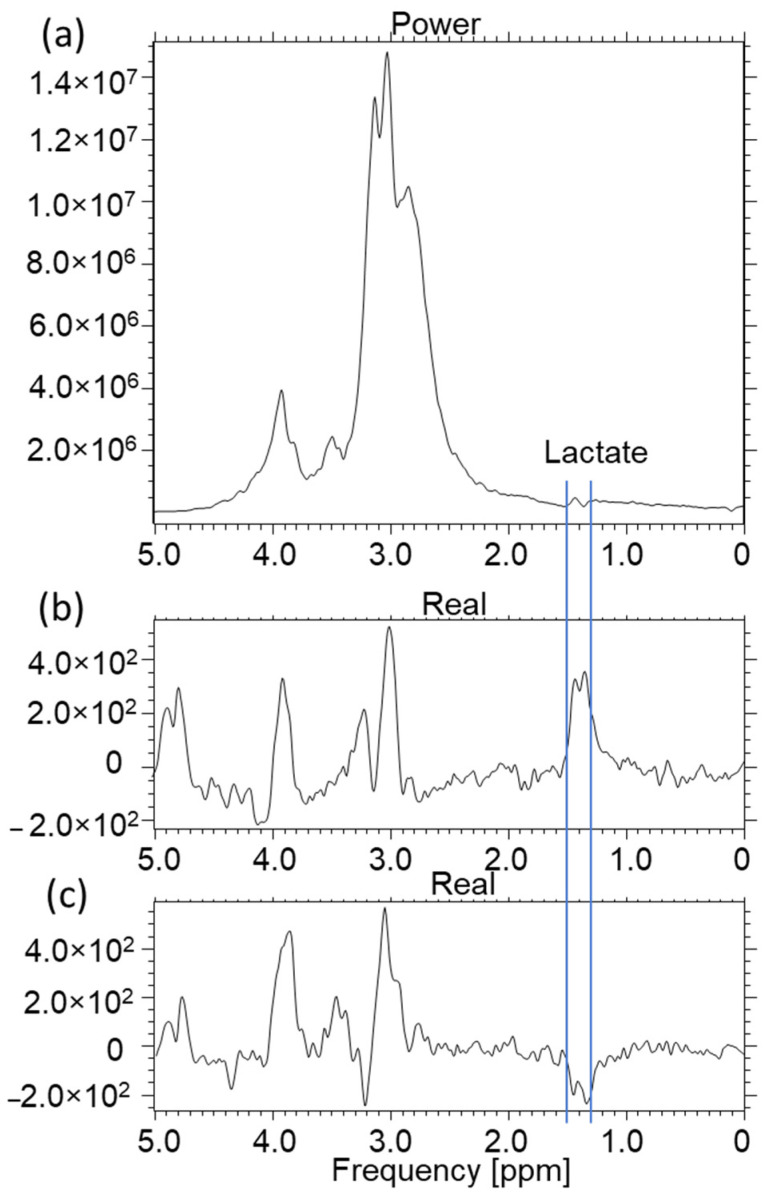
Spectroscopy of the CRL2314 cell culture with marked locations for the lactate peaks, (**a**) spectrum in Power mode TE = 144, TR = 4000 ms, NEX = 8, total number of scans: 128, sequence name: PROBE-P, (**b**) component of the Real spectrum TE = 288 ms, TR = 4000 ms, NEX = 8, total number of scans: 128, sequence name: PROBE-P, (**c**) component of the Real spectrum TE = 144 ms, TR = 4000 ms, NEX = 8, total number of scans: 128, sequence name: PROBE-P. The test performed with the experimental coil shown in Figure 2. Voxel size for all spectra: 8 × 8 × 8 mm (own study).

Figure 9 shows the results of the MRS spectroscopy for the CRL2314 lung cancer cells in cell culture. The spectrum was obtained from the coil described in this paper.

Based on the research using the MR 1.5 T system for the A549 cell culture, it can be said with certainty that this system is suitable for conducting analyses over a limited range. This truncation results from the properties of the phenomena themselves, which is characteristic of magnetic resonance spectroscopy. The tests can be carried out many times during the growth of a cell colony, and it is a non-destructive test. Other distribution tests, for example, NMR, which requires the complicated preparation of biological material, ends with, in essence, the death of cells and a necessity to dispose of the culture. Of course, it should be noted that MRS tests with low values for the magnetic field can in no way be used to identify unknown metabolites. However, this type of research has an undeniable advantage over others, as it is much more accurate and precise for research, and it allows the cell culture to be kept alive.

## 3. Discussion

This paper deals with the goal of detecting lactate in cell cultures by using ^1^H MRS.

The selected object is surrounded by a huge concentration of aqueous media solution. This makes the water signal a huge disruptor. In order to eliminate the H_2_O component from the spectra, it was necessary to use the spectroscopic sequence to suppress the water signal. The most frequently used method is CHESS (chemical shift selective saturation). Clinical systems most often use triple water suppression to better eliminate the water. There are some variants of CHESS and its modifications, namely WET (water suppression enhanced through T_1_ effects) [9], MOIST (multiple optimizations insensitive suppression train), SWAMP (suppression of water with adiabatic-modulated pulses) [10], and VAPOR (variable power radiofrequency pulses with optimized relaxation delays) [11]. It is not possible in practice to observe spectra for TE < 25 ms. The STEAM method has an advantage in terms of the possibility of observing the spectra of metabolites with short T_2_ times [9,10,11,12,13].

**Figure 10:**
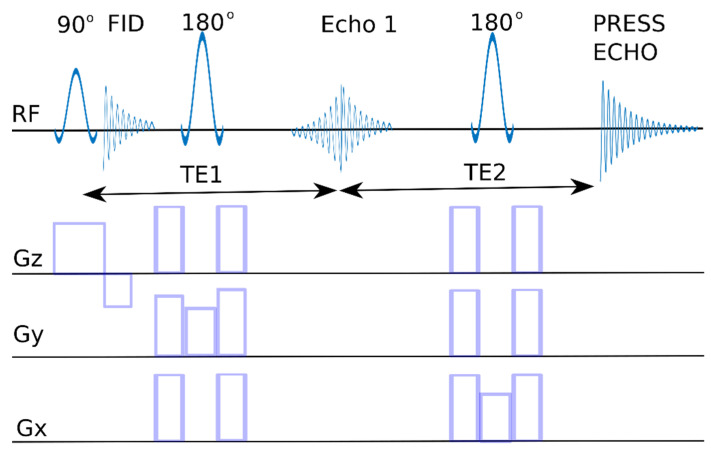
PRESS sequence diagram. RF- signal, with resonance frequency, Gx, Gy, Gz—gradient pulses in the X, Y, Z axes, 90°, 180°—pulse inverting the magnetization vector by an angle of 90° and 180°, respectively, PRESS ECHO—recorded signal, TE—echo time.

**Figure 11:**
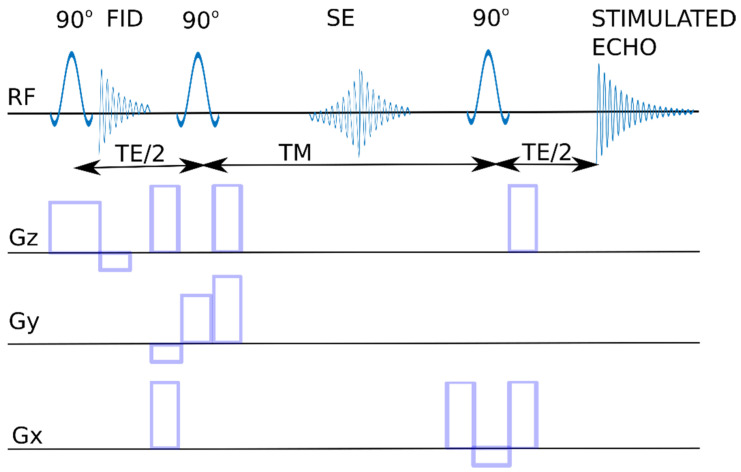
STEAM sequence diagram. RF- resonant frequency signal, Gx, Gy, Gz—gradient pulses in the X, Y, Z axes, 90°—pulse inverting the magnetization vector by an angle of 90°, STIMULATED ECHO—recorded signal, TE—echo time, TM—mixing time.

When talking about MRS spectroscopy, it is impossible not to mention the homogeneity of the field, which is the most important parameter of the MR system. In general, this parameter in MRS is much worse than in NMR systems. It is this factor that determines the resolution of the spectroscopic acquisition, and the differentiation of individual signals depends on it. In medical research, due to the need to minimize the examination time for the patient, automatic shimming is most often used, which ensures optimal values for examination, e.g., of the head. It should be added that many systems allow for the manual tuning of the magnet, which often allows for better spectra. “Medical” magnets have a low resonance frequency, so a large part of the signals overlap, making the analyzed image more difficult. These are the basic problems of low-yield systems, and, in practice, it is difficult to identify complex chemical compounds in these systems. Below are the presented two pulse sequences used for water suppression (Figure 10 and Figure 11). In both cases, the area under study is selected by overlapping the magnetic fields from three axes derived from three gradients. Only the area which experiences the simultaneous action of these three magnetic fields superimposed on the B0 field is the source of the spectroscopic signal. For the PRESS sequence (point resolved spectroscopy), a 90°–180°–180° spin echo sequence is necessary, while for the STEAM sequence (stimulated echo acquisition mode), 90°–90°–90° pulses are used.

It should be noted that MRS is successfully used in medicine and the effect of the inverted resonance signal allows for the clear distinction of lactate from lipids. The work is the initial part of a larger project in which researchers attempt to identify as wide a group of metabolites as possible in cell cultures using the medium-field MR system. The use of a 1.5 T field induction magnet generates many problems related to the separation of metabolites or chemical compounds included in the medium. The overlapping of the spectra makes these problems difficult to solve. In addition, a big problem is the low homogeneity of the B0 magnetic field present in clinical systems compared to NMR systems. In addition to the increased width of the spectral fringes, this also results in worse water attenuation. The noise of the B0 field is another important parameter that determines the success and quality of the spectroscopic examination [1]. The substances that can be detected by the ^1^H MRS method also include alanine (1.48 ppm), ethanol (1.2 ppm), glycine (3.55 ppm), glucose (4.63 ppm), glutathione (3.77 ppm), ascorbic acid (3.73 ppm, 4.01 ppm and 4.50 ppm), mannitol (3.8 ppm), serine (3.98 ppm, 3.94 ppm and 3.83 ppm), and valine (1.1 ppm). However, due to their low concentration in the examined tissues and the fact that they do not provide the necessary diagnostic information, the concentrations of these substances are not assessed in standard procedures. Peak analysis for these substances is performed in specific scientific studies, wherein researchers specifically focus on the presence or changes in the concentrations of specific substances. In the case of pathological processes within the neoplastic tissue, there are changes in the concentration of individual compounds, which is visible in the form of changes in the height of the peaks in the diagram. For example, an increase in lactate concentration is a marker of anaerobic metabolism; an increase in the lipid peak indicates the breakdown of cell membranes. The MRS method is a limited method in terms of sensitivity. It is undoubtedly true that MRS in cell culture studies cannot serve to identify unknown compounds. This part of the research is invariably reserved for high-field NMR systems as well as other test methods. The development of electronics, high-speed multi-bit analog-to-digital converters, along with the advances in the design and production of broadly understood “analog electronics”, combined with the development of digital methods of data analysis, significantly contributed to the improvement in this area. However, the analysis of data obtained as a result of spectroscopic scanning is necessarily limited to those metabolites, the concentration of which allows for their visualization in this method [8,9].

The basic problem is to match the transmitting-receiving coils, which can be used in the tested structures and are objects with a volume not exceeding 2 mL. During the implementation of the works, it turned out that it was necessary to design several variants of the receiving coils for imaging. These circuits were RLC circuits tuned to the resonance frequency specific to the ^1^H nuclei for the 1.5 T field, namely 63.885 MHz. These dedicated coils were matched to the geometry of the given objects. In the case of spectroscopic examinations, the receiving coil was a solenoid type. This solution provided very good conditions related to the homogeneity of the B1 field. Each of these receiving circuits was precisely tuned using the DSA 815 spectrum analyzer from RIGOL. This analyzer, which includes a tracking generator in its design, measures the characteristics of the analyzed system in the frequency range from 9 kHz up to 1.5 GHz. Such a range of analysis allows for a thorough examination of the receiving system.

Lactate measurements at 1.5 Tesla provide an elevated level of the signal from lactate, showing an increase in anaerobic metabolism caused by (e.g., cerebral hypoxia, ischemia, and metabolic disorders) the presence of tissue that has poor metabolism (e.g., cysts and tumors).

Analyzing the literature, it is perfectly clear that the problems mentioned here are overcome by the use of strong magnetic fields with induction, e.g., 7 T, 11 T, or more, and therefore much higher resonant frequencies. Unfortunately, the limitations of medicine, safety, and the design conditions of the scanners prevent their wide application. It is also important that medium-field MR scanners are equipped with many scientific units.

Nevertheless, MRS spectroscopy yields a number of answers. Signal analysis, taking into account the previously acquired knowledge about the shape of the signals corresponding to the individual metabolites, can lead to satisfactory quantification results. It should be added that the method depends on many factors, which are the properties of the tested sample as well as the environment in which the test takes place. The most important of them are temperature, solution concentration, type of solvent, electron density, presence of hydrogen bonds, pH factor, and sample contamination.

The obtained results allow us to conclude that an appropriate approach to conducting this diagnosis will enable the identification of a certain group of metabolites characteristic of neoplastic diseases. The vast majority of tumor processes, both primary and metastatic, are associated with an increase in glucose concentration. This results in increased glucose metabolism. Glucose metabolism (to lactate) leads to microenvironmental acidosis, which, in turn, increases the resistance to this factor. Increased immunity promotes the greater proliferation and invasion of the neoplastic cells [13,14]. Cancer cells consume a disproportionate amount of nutrients compared to other cell types. This fact is partly due to the oxygen glucose.

The precursor research on cellular metabolism (Otto Warburg) noted that neoplastic cells generate energy mainly through glucose in the intracellular fluid, and this phenomenon is independent of the amount of available oxygen. This discovery is called the “Warburg Effect.” Healthy cells also produce energy through glucose, but this happens when the amount of oxygen is low [15]. Additional information on this topic can be found in [16,17,18]. Additionally, it has been proven that in various neoplastic processes, the levels of lactate, alanine, and lipids increase. The subject related to lipids in cell cultures has been discussed in many publications, among which it is worth mentioning, for example, the works of [19,20,21,22]. An extensive discussion on glutamine metabolism is included in [18,23]. It is known that the concentration of total Glx (glutamate (Glu) + glutamine (Gln)) but also choline, lactate, and lipids is higher in high-grade gliomas compared to low-grade disease. It should be mentioned that the differentiation of Glu and Gln is much more difficult due to the overlapping of some spectra [24]. The authors indicate that cancer cells show a greater need for glutamine. Interference with glutamine metabolism shows great potential for cancer therapy. Researchers [25,26,27,28] report that metabolites containing phosphocholine and glycerophosphocholine are associated with the malignancy of the neoplastic process. Choline (Cho) is a marker of the severity of the neoplastic process in the studied area, and its metabolism is a characteristic feature of carcinogenesis and tumor growth, which increase the levels of phosphocholine (PCho) and glycerophosphocholine (GPC) [28]. These three compounds, defined as total choline (tCho) in medium-field MR systems, give resonance signals closely located to each other, which significantly hinders their separation. The development of MRS spectroscopy in recent years has enabled the development of choline research [23]. Creatine (Cr) indicates the energy metabolism in the cell. Its reduction is a sign of cell necrosis or death.

The above-mentioned metabolites are of interest to the authors of this study. The use of the mid-field MRS system is fully justified from the perspective of the further development of in vivo MRS research. The analysis of cell cultures allows for the elimination of many factors occurring in vivo and overlapping with the test result. In a controlled culture environment, only the response of the cells themselves to external conditions remains. The use of medium-induction systems in the in vitro field will also make it easier to transfer the results of the analyses to the in vivo field. At present, routine research in high-field systems is not very popular, so the proposed solution by the authors of the paper seems to be entirely justified. The relative ease of adaptation of the receiving systems (coils to the needs of breeding) allows for research to be conducted to a higher quality than with the use of factory-supplied coils. The effect of different TEs on the detection of metabolites with more complex J-evolution dynamics, such as glutamate, glutamine, and myo-inositol, is an active area of investigation [29,30].

The lactate peak is visible in the ^1^H MRS spectrum in the process of anaerobic metabolism and occurs in small amounts in healthy tissue, which makes it difficult to isolate because it gives a signal at the noise level. The visible Lac peak indicates the presence of anaerobic metabolism in the case of poor metabolism tissues (e.g., cancer). To assess the presence of lactate in the tissue, a TE time in the range of 135–144 ms is used because then, the lactate peaks are inverted so that these peaks are visible on the spectogram below the baseline and are not obscured by noise.

When analyzing their signals recorded separately, one can notice the striking similarity between the peaks and their immediate vicinity, which causes difficulties in the separation of these signals. An extensive discussion of glutamine metabolism has been included in [31]; there, the authors point out that cancer cells show a greater need for glutamine. The interference with glutamine metabolism shows great potential associated with cancer therapy.

Oncogenesis and tumor progression are closely related to choline metabolism, which has been extensively studied. The ^1^H MRS spectroscopy of choline-containing compounds shows them in the spectrum (as a peak) in the region of approximately 3.2–3.3 ppm. In high-resolution spectroscopy, choline (Cho—N (CH_3_)_3_—3.185 ppm, CH_2_—3.501 ppm, CH_2_—4.054 ppm), phosphocholine (PCho—N (CH_3_)—3.208 ppm, CH_2_—3.641) can be visualized in this area (ppm, CH_2_—4.28 ppm), and for glycerophosphocholine: (GPC—N (CH_3_)_3_—3.212 ppm, CH_2_—3.605 ppm, 3.659 ppm, 3.672 ppm, 3.871 ppm, 3.946 ppm, 4.312 ppm, CH—3.903 ppm). As the peaks of choline (Cho), phosphocholine (PCho), and glyerophosphocholine (GPC) are very close to each other in the magnetic resonance spectrum and form a single signal in systems with lower spectral resolutions, the term total choline (tCho) has also been introduced.

In [32], the researchers reported that metabolites containing phosphocholine and glycerophosphocholine are associated with the malignancy of the neoplastic process. Additionally, they informed that these compounds have been proposed as biomarkers of tumor progression. ^1^H MRS spectroscopy and total choline signal can be used as an indicator of tumor response to chemotherapy or radiotherapy [33,34].

As already mentioned, choline is a marker of the increase in the neoplastic process in the area studied, and its metabolism is a characteristic feature of carcinogenesis and tumor growth, which increases the level of phosphocholine and glycerophosphocholine [35]. However, Cho is not the only marker that can be imaged in MRS. Creatine (Cr) indicates the energy metabolism of cells. Its reduction is a sign of necrosis or cell death.

In order to detect increased anaerobic glucose, it is necessary to test the level of lactate. Increasing the level of this metabolite informs the investigator of hypoxemia.

In the early phases of research into the metabolism of neoplastic cells, an increased level of phosphorocholine (PCho) was interpreted as the end of a high rate of cell proliferation. Only the development of MRS spectroscopy in recent years has made it possible to discover the metabolic trait of the choline phenotype [36]. Its characteristic feature, which is the increased activity and expression of the ChoK-alpha enzyme, makes it suitable for therapy. There has been a significant increase in MRS and NMR research on the cellular metabolome (Table 2). The table below summarizes examples of research regarding cellular composition detected by MRS.

MRS enables the early detection of neoplastic changes. The hardware based on physical instrumentation and new pulse sequences, and the software, based on innovative postprocessing methods for MRS, are improving. It is hoped that MRS will be on optimal choice in the selection of diagnostic methods and will best support medical research and hospitals.

## 4. Materials and Methods

### 4.1. The Hardware

^1^H MRS was recorded using an OPTIMA 360MR. The MRS examination is demanding both in terms of equipment and preparation. The OPTIMA 360MR system is a clinical apparatus that perfectly performs tasks related to the diagnosis of patients. The receiver coils that are included in the equipment of the OPTIMA 360MR perfectly illustrate the human body. However, this system was not designed for cell culture research, e.g., MRS. The large sizes of the coils which the system uses do not allow for the accurate imaging and spectroscopy of such small structures.

**Figure 12:**
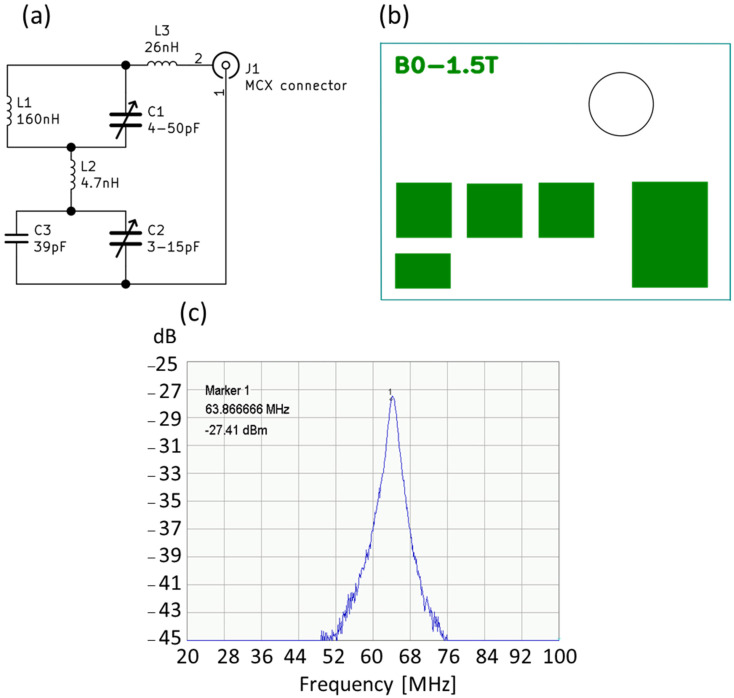
(**a**). Diagram of the receiving part of the coil, (**b**) arrangement of solder pads on the PCB, (**c**) receiving coil frequency response.

Due to the small size of the cell samples, it was necessary to develop a receiving coil for MRS purposes only, using the OPTIMA 520 MR. The designed coil acquired the RF signal as a single-channel solenoid coil. Photographs of the coil are presented in Figure 12a–c. The receiving circuit for MRI signal acquisition is shown. This solution allows for the significant simplification of the designed circuit for a specific application; the Ependorf tube is used to record the signal.

Figure 12a shows the electrical diagram of the receiving part of the designed coil. The construction uses passive electronic elements that can work in the magnetic field. Such elements are made entirely without the use of ferromagnetic materials. The values of the L1 coil were measured after it was wound and adjusted to the shape of the Eppendorf tube, with the remaining elements selected in such a way as to obtain the best possible signal. The values indicated in the diagram, in particular, for the coils, were determined using an RLC meter (type: U1733C prod. Agilent). This device measures the value of RLC elements using the bridge method, which ensures high accuracy.

**Figure 13:**
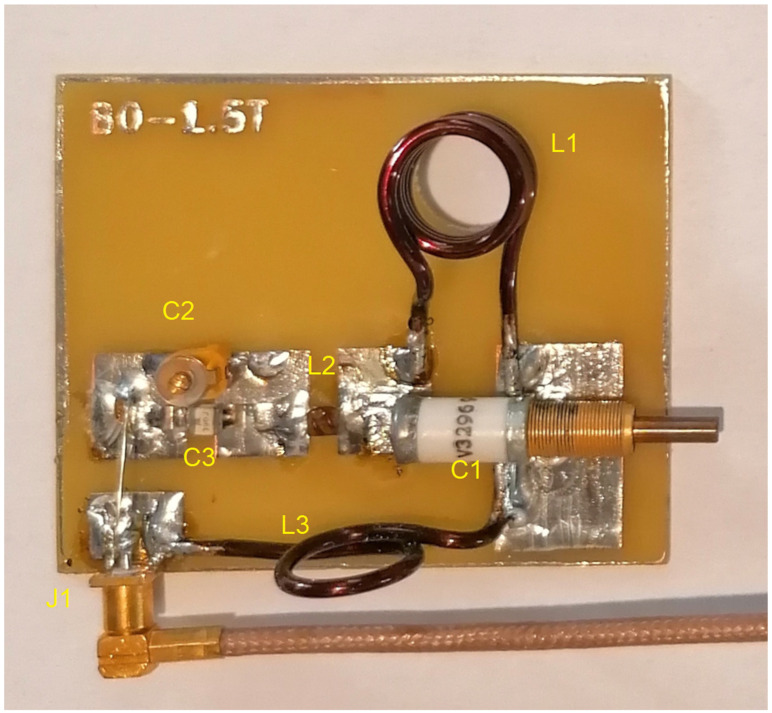
The appearance of the assembled receiver. L1, L2, L3—induction coil, C1, C2, C3—capacitor, J1—MCX connector (own elaboration).

The entire circuit assembled on the PCB shown in Figure 12a,b was connected to the factory-supplied single-wire coil adapter using a 50 ohm coaxial cable. After assembling the electronic circuit, it was tuned using the RIGOL spectrum analyzer, type: DSA815. This analyzer has a wide band of generated, recorded, and analyzed RF signals, ranging from 8 kHz up to 1.5 GHz. Such a band guarantees the examination of the system for use in the MR system. Figure 12c shows the spectrum of the RF signal in the tested 80 MHz bands. The bandwidth of the coil is about 2.9 MHz (−3 dB), which guarantees no influence from this parameter on the received RF signal. Figure 13 presents the assembled receiver. 

### 4.2. The Software

The acquisition of ^1^H MRS was carried out using echo times (TE), ranging from 27 ms to 328 ms, with a step of about 10 ms. The protocol, with the use of the CHESS (CHEmical Shift Selective saturation) technique, has been developed to remove the unwanted signal of water [48]. CHESS is based on an excitation pulse with a selective frequency and an inversion angle of 90°. This pulse is followed by a gradient pulse, which de-phases the spin system. After this treatment, the magnetization of the undesirable signal, in this case, water, disappears. Hydrogen nuclei, with a different chemical shift than those for which the pulse described earlier, was intended, remain intact. Three CHESS pulses were required for spectroscopic application. This is due to the insufficient attenuation of water via this method for MR signal acquisition.

### 4.3. Samples

The next step in this analysis was to examine the culture medium. RPMI 1640 cell culture medium, developed at Roswell Park Memorial Institute, formulated by ATCC (ATCC 30-2001) was purchased in a liquid form. To obtain a complete growth medium suitable for cell culture, Fetal Bovine Serum (FBS, ATCC 30-2020) was added to the base medium for a final concentration of 10%, and an antibiotic, Penicillin, was added. The complete medium was prepared from the ingredients heated to 37 °C in the following order: 3 mL of FBS and 300 µL of Penicilin antibiotic were added to 27 mL of RPMI-1640 medium. The pH of the medium was pH = 8. The test was carried out in the absence of an external standard at a temperature of 22 °C. The signal analysis was carried out in the SAGE7.7.1 program, which allows for the introduction of a correction related to the temperature. The tested voxel, with dimensions of 8 mm × 8 mm × 8 mm, contained approximately 512 mm^3^ of fluid.

To measure the ^1^H MRS of the lactate peak, as a function of TE, an aqueous solution of lactate, with a concentration of 0.1% in 10 mL of water, was prepared. Lactate solution, with an initial concentration of 80%, was obtained from the manufacturer, CHEMPUR (Piekary Sląskie, Poland).

The measured samples of media or lactate solution had a volume of 1.5 mL and were placed in an Eppendorf tube, for which the coil presented in Figure 12b was designed.

## 5. Conclusions

In conclusion, it should be said that MRS, with the use of medium-field magnetic resonance systems, has significant potential for the study of cancer metabolism. MRS provides in vivo chemical information about the metabolic profile of tissue that is not accessible by any other means using the currently available experimental methods. The need to develop new forms of drugs, along with the development of molecular, immunological, biochemical, and physicochemical techniques in basic and preclinical research, has led to the possibility of obtaining information using MRS.

This study provides a template for metabolomics studies on lactate in cells and provides the possibility of collecting data from cancer tissue without sample preparation.

## Figures and Tables

**Table 1:** The chemical composition of RPMI-1640.

Component	Concentration [g/L]
Inorganic salts	
Ca(NO_3_)_2_ × 4H_2_O	0.1
MgSO4 (anhyd)	0.04884
KCl	0.4
NaCl	6.0
Na_2_HPO_4_ (Anhyd)	0.8
Amino acids	
L-Arginine (free base)	0.2
L-Asparagine (anhyd)	0.05
L-Aspartic Acid	0.02
L-Cystine × 2HCl	0.0652
L-Glutamic Acid	0.02
L-Glutamine	0.3
Glycine	0.01
L-Histidine (free base)	0.015
Hydroxy-L-Proline	0.02
L-Isoleucine	0.05
L-Leucine	0.05
L-Lysine × HCl	0.04
L-Methionine	0.015
L-Phenylalanine	0.015
L-Proline	0.02
L-Serine	0.03
L-Threonine	0.02
L-Tryptophan	0.005
L-Tyrosine × 2Na × 2H_2_O	0.02883
L-Valine	0.02
Vitamins	
D-Biotin	0.0002
Choline Chloride	0.003
Folic Acid	0.001
myo-Inositol	0.035
Niacinamide	0.001
p-Amino Benzoic Acid	0.001
D-Pantothenic Acid × ½Ca	0.00025
Pyridoxine·HCl	0.001
Riboflavin	0.0002
Thiamine × HCl	0.001
Vitamin B-12	0.000005
Other	
D-Glucose	2.0
Glutathione (reduced)	0.001
Phenol Red × Na	0.0053
NaHCO_3_	2.0

**Table 2:** Review of the literature.

MR System/Research System	Test Cells	Type of Cells	Source	Medium	Number of Cells	Analyzed Biomarker and/or Its Function
400.14 MHz, Avance (Bruker, AG, Darmstadt, Germany)	MCF-7	breast cancer	(Grande, 2006) [37]	RPMI 1640	~5 × 10^6^	GSH metabolism (γ-glutamylcysteinylglycine tripeptide, glutathione)
400.14 MHz, Avance (Bruker, AG, Darmstadt, Germany)	TG98	malignant glioma	(Grande, 2006) [37]	RPMI 1640	~5 × 10^6^	GSH metabolism (γ-glutamylcysteinylglycine tripeptide, glutathione)
600 MHz, Varian	MCF-7	breast cancer	(Ward, 2013) [38]	DMEM	5 × 10^6^	Phosphocholine (PC)
500 MHz, 600 MHz (Varian/Agilent Palo Alto, CA, USA)	PC3	prostate cancer	(Ward, 2013) [38]	DMEM low in glucose	-	Glucose → lactate; choline-containing metabolites: (tCho, consisting of choline, phosphocholine (PC) and glycerophospho choline (GPC))
500 MHz, 600 MHz (Varian/Agilent Palo Alto, CA, USA)	MCF-7	breast cancer	(Lodi, 2014) [39]	DMEM low in glucose	-	Glucose → lactate; choline-containing metabolites: (tCho, consisting of choline, phosphocholine (PC) and glycerophospho choline (GPC))
500 MHz, 600 MHz (Varian/Agilent Palo Alto, CA, USA)	A375	melanoma cells	(Lodi, 2014) [39]	DMEM high in glucose	-	Glucose → lactate; choline-containing metabolites: (tCho, consisting of choline, phosphocholine (PC) and glycerophospho choline (GPC))
400.14 MHz, Bruker, AG, Darmstadt, Germany	MCF-7	breast cancer	(Rosi, 2007) [40]	RPMI 1640	20 × 10^6^	glutamate (Glu), glutamine (Gln), glutathione (GSH)
400.14 MHz, Bruker, AG, Darmstadt, Germany	HeLa	cervical cancer	(Rosi, 2007) [40]	EMEM	20 × 10^6^	glutamate (Glu), glutamine (Gln), glutathione (GSH)
500 MHz INOVA (Varian, Santa Clara, CA, USA)	MCF-7	breast cancer	(Brandes, 2010) [41]	Eagle		phosphocholine (PC)
7 T Bruker BioSpec Avance 70/20	MCF-7	breast cancer	(Jensen, 2010) [42]	Earle	1 × 10^7^	Choline (Cho)
9.4 T Bruker Horizontal Bore Small Animal Scanner (Bruker BioSpin Corp.)	MCF-7	breast cancer	(Wijnen, 2014) [43]	HBSS	2 × 10^6^	metabolites phospholipids, phosphoethanolamine (PC), phosphoethanolamine (PE), glycerophosphocholine (GPC) and glycerophosphoethanolamine (GPE)
9.4 T Bruker Horizontal Bore Small Animal Scanner (Bruker BioSpin Corp.)	MDA-MB-231	breast cancer (highly aggressive)	(Wijnen, 2014) [43]	HBSS	2 × 10^6^	metabolites phospholipids, phosphoethanolamine (PC), phosphoethanolamine (PE), glycerophosphocholine (GPC) and glycerophosphoethanolamine (GPE)
400 MHz Bruker; AM400 WB	MCF-7	breast cancer	(Le Moyec, 1992) [44]	Eagle (DMEM, Gibco), 10% fetal calf serum	-	lipids
400 MHz Bruker; AM400 WB	T47D	breast cancer	(Le Moyec, 1992) [44]	Eagle (DMEM, Gibco), 10% fetal calf serum	-	lipids
400 MHz Bruker; AM400 WB	ZR-75-1	breast cancer epithelial cell	(Le Moyec, 1992) [44]	Eagle (DMEM, Gibco), 10% fetal calf serum	-	lipids
400 MHz Bruker; AM400 WB	SKBR3	breast cancer	(Le Moyec, 1992) [44]	Eagle (DMEM, Gibco), 10% fetal calf serum	-	lipids
400 MHz Bruker; AM400 WB	MDA-MB231	breast cancer (highly aggressive)	(Le Moyec, 1992) [44]	Eagle (DMEM, Gibco), 10% fetal calf serum	-	lipids
-	K562	myeloid leukemia	(Le Moyec, 2000) [45]	RPMI 1640	-	lipids
500 MHz, Variaan; Unity Inova	K562	myeloid leukemia	(Mannechez, 2005) [46]	RPMI	1 × 10^7^	lipids
400.14 MHz Bruker, Avance	MCF-7	breast cancer	(Grande, 2007) [47]	RPMI 1640		glutathione (GSH)
400.14 MHz Bruker, Avance	HeLa	cervical cancer	(Grande, 2007) [47]	EMEM	20 × 10^6^	glutathione (GSH)

## Data Availability

Not applicable.

## Abbreviations

MRS	magnetic resonance spectroscopy
NMR	nuclear magnetic resonance
Glu	glutamate
Gln	glutamine
Glx	Glu + Gln
^1^H	hydrogen
Cho	choline
PCho	phosphocholine
GPC	glycerophosphocholine
tCho	total choline
Cr	creatine
RF	radio frequency electromagnetic wave
RPMI 1640	a medium for cell culture
MCF-7	a breast cancer cell line
A549	Lung cancer cell line
TE	echo time
TR	repetition time
PCB	printed circuit board
